# Constructing maps between distinct cell fates and parametric conditions by systematic perturbations

**DOI:** 10.1093/bioinformatics/btad624

**Published:** 2023-10-13

**Authors:** Ruoyu Tang, Xinyu He, Ruiqi Wang

**Affiliations:** Department of Mathematics, Shanghai University, Shanghai 200444, China; Department of Mathematics, Shanghai University, Shanghai 200444, China; Department of Mathematics, Shanghai University, Shanghai 200444, China; Newtouch Center for Mathematics of Shanghai University, Shanghai University, Shanghai 200444, China

## Abstract

**Motivation:**

Cell fate transitions are common in many developmental processes. Therefore, identifying the mechanisms behind them is crucial. Traditionally, due to complexity of networks and existence of plenty of kinetic parameters, dynamical analysis of biomolecular networks can only be performed by simultaneously perturbing a small number of parameters. Although many efforts have focused on how cell states change under specific perturbations, conversely, how to infer parametric conditions underlying distinct cell fates by systematic perturbations is less clear and needs to be further investigated.

**Results:**

In this article, we present a general computational method by integrating systematic perturbations, unsupervised clustering, principal component analysis, and fitting analysis. The method can be used to to construct maps between distinct cell fates and parametric conditions by systematic perturbations. In particular, there are no needs of accurate parameter measurements and occurrence of bifurcations to establish the maps. To validate feasibility and inference performance of the method, we use toggle switch, inner cell mass, and epithelial mesenchymal transition as model systems to show how the maps are constructed and how system parameters encode essential information on cell fates. The maps tell us how systematic perturbations drive cell fate decisions and transitions, and allow us to purposefully predict, manipulate, and even control cell states. The approach is especially helpful in understanding crucial roles of certain parameter combinations during fate transitions. We hope that the approach can provide us valuable information on parametric or perturbation conditions so some specific targets, e.g. directional differentiation, can be realized.

**Availability and implementation:**

No public data are used. The data we used are generated by randomly chosen values of model parameters in certain ranges, and the corresponding parameters are already attached in supplementary materials.

## 1 Introduction

One of the most significant challenges in biology is to elucidate the relationships between various cell fates and their decision-making mechanisms (Rukhlenko *et al.* 2022). Most biological processes involve precise cellular state decisions and transitions controlled by underlying molecular regulatory processes and exogenous and endogenous stimuli such as ligands, inhibitors, growth factors, or drugs. Modeling and predicting the effects of perturbations, e.g. epithelial mesenchymal transition (EMT) after exogenous TGF-β treatment (Tian *et al.* 2013), are key tasks both in biology and mathematics. Fundamentally, an accurate understanding effects of systematic perturbation may help us predict cell state transitions and purposefully control them. Therefore, constructing maps between cell fates and their perturbation mechanisms is becoming more and more important, by which we can screen targeted perturbations and further convert cell fate decisions (Caldera *et al.* 2019).

Modeling and predicting effects of perturbations are often realized by bifurcation analysis mathematically. Especially, it is powerful when the number of perturbed parameters is less (Kuznetsov 1995). Bifurcation theory can be also used to estimate synergism between combinatorial perturbations (Luo *et al.* 2021). However, due to the high dimension and complexity of molecular networks, when more parameters are perturbed simultaneously, it becomes difficult to perform bifurcation analysis. Luckily, rapid development of experimental and computational biology approaches have allowed us to address this question (Molinelli *et al.* 2013, Katebi *et al.* 2020, Ji *et al.* 2021).

Many efforts have focused on how a cell state changes fates after perturbations, e.g. cell state transitions determined by Waddington’s landscape (Wang *et al.* 2011) and lineage tree (Hormoz *et al.* 2016), bifurcation analysis (Wang *et al.* 2011, Huang and Wang 2020), random parametric perturbations (Huang *et al.* 2017, 2018), or network topology screening with random sampling of parameter space (Yao *et al.* 2011, Fu *et al.* 2012). Classification of regions in parameter spaces of nonlinear dynamical systems with different qualitative behavior and parameter-phenotype mapping are important topics both in mathematics and in biology (Hasenauer *et al.* 2010, 2012), however, few research focuses on such mappings and cell fate inference under certain parametric conditions through computational and statistical analysis. There still exists a critical gap on how systematic perturbations drive cell fate decisions and transitions, which allows us to purposefully predict, manipulate, and even control cell states.

To address this issue, the purpose of this article is to present a general computational approach to construct maps between distinct cell fates and parametric conditions by systematic perturbations. The approach integrates randomly systematic perturbations, unsupervised clustering, principal component analysis (PCA), and fitting analysis. Randomly systematic perturbations are performed to generate steady state datasets for clustering so as to form robust cell categories. Clustering has been widely sued to distinguish different cell states in dataspace (Wang *et al.* 2014, Huang *et al.* 2017, Katebi *et al.* 2020). PCA is performed mainly to reduce dimension of steady state space because it is expected that important cell fate features are lower dimensional. Next, we adopt fitting analysis, i.e. partial least-squares (PLS) regression (Geladi and Kowalski 1986, Boulesteix and Strimmer 2007) and confidence ellipse, for classification and prediction, although other more complex strategies, e.g. support vector machines (SVM) (Rukhlenko *et al.* 2022), can also be applied.

To validate feasibility and prediction performance of the approach, we use toggle switch (TS) (Gardner *et al.* 2000), inner cell mass (ICM) (Bessonnard *et al.* 2014), and EMT (Tian *et al.* 2013) as model systems to show how the maps are constructed and how system parameters encode essential information on cell fates. The maps revealed by the approach are largely consistent with ICM and EMT related perturbation experimental observations in literatures (Xu *et al.* 2009, Tian *et al.* 2013, Bessonnard *et al.* 2014, Cook and Vanderhyden 2020). Systematic perturbations provide more insight into information contained in parametric conditions. In addition, importance of each parameter in determining each cell fate can be further assessed by estimating the variable importance in projection (VIP) scores (Saeys et al. 2007).

## 2 Materials and methods

The approach starts by randomly perturbing all parameters to generate steady state data, which are then clustered to identify distinct but robust state categories. Due to high dimension of the data, dimensionality reduction is performed by PCA so that fitting analysis can be carried out. Finally, mapping between cell fates and parametric conditions can be established by the classification criterion estimated by the fitting analysis. Detailed implementation process is made as follows.

### 2.1 Step 1: systematic perturbations to generate datasets

For a network, the parameters in its corresponding dynamical model are randomly chosen. It is assumed that each parameter obeys the uniform distribution in an appropriate interval. And each parameter set corresponds to one or more datasets of stable steady states. For each parameter set, we numerically solve the dynamical model with some random initial conditions and repeat sometimes so as to minimize interference from initial conditions and attraction domains, etc., and thoroughly identify all possible steady state solutions, based on which we further determine the number of distinct stable states.

- For a system with state variables $x={\left(x_{1},x_{2},\ldots ,x_{m}\right)}^{T}\in R^{m}$ and parameters $p={\left(p_{1},p_{2},\ldots ,p_{s}\right)}^{T}\in R^{s}$, *n* randomly chosen parameter sets constitute a matrix $P_{n\times s}$

$$P_{n\times s}=\left(\begin{array}{cccc} p_{11} & p_{12} & \cdots & p_{1s}\\ p_{21} & p_{22} & \cdots & p_{2s}\\ \vdots & \vdots & \ddots & \vdots \\ p_{n1} & p_{n2} & \cdots & p_{ns} \end{array}\right).$$

At *n* randomly chosen parameter sets, steady state sets of the dynamical system corresponding to $P_{n\times s}$ is

$$X_{q\times m}=\left(\begin{array}{cccc} x_{11} & x_{12} & \cdots & x_{1m}\\ x_{21} & x_{22} & \cdots & x_{2m}\\ \vdots & \vdots & \ddots & \vdots \\ x_{q1} & x_{q2} & \cdots & x_{qm} \end{array}\right).$$

Generally speaking, q≥n because multiple steady states may exist at a certain parameter set. For the datasets of parameters and stable steady states, we can divide them into two categories, i.e. monostability and multistability. We use them as the class label of training sets and perform supervised kernel support vector machines (KSVM) classification. The test accuracy is around 98.6% and 87.6% for TS and EMT network through KSVM, respectively. For each parameter set given within the perturbation intervals, we can predict whether it is monostable or multistable under the given parameter set.

### 2.2 Step 2: Mapping under monostable condition

We first consider the case of monostability, i.e. only one stable steady state for a given parameter set.

- Divide the data matrix of monostable states, $X_{q^{\prime }\times m}$ with $\left(q^{\prime }\leq q\right)$ into different categories, i.e. cell fates, according to their biological information or clustering analysis.
- Steady state data are generally high-dimensional, and therefore hard to interpret intuitively. Dimensionality reduction is performed by linear PCA. When strong nonlinear relationship among data exists, nonlinear dimensionality reduction techniques like kernel principal component analysis can be utilized. Generally, data will be limited to the first few PCs which contribute much for data exploration. In our examples, we use only PC1, which contributes sufficient information (approximately 90%). For the case where PC1 contains insufficient information, more PCs need to be incorporated.
- To infer relationship between response variable, i.e. the first PCs of stable steady state variables, and explanatory variables, i.e. parameters, fitting analysis is applied to estimate the mathematical relationship for explaining *x* in terms of *p* or vice versa. The fitting analysis provides us classification criteria and prediction ability so that we can construct maps between cell fates and certain parameter combinations. Note that the choice of resolution determines the number of clusters, i.e. the number of cell fates.
- To verify the classification performance of the approach, for all clusters, 70% of the steady state sets, i.e. $X_{n_{1}\times m}$, and parameter sets related to $X_{n_{1}\times m}$, i.e. $P_{n_{1}\times s}$, form the training sets. Relatively, the remaining 30% act as the test sets to validate classification efficiency of the fitting analysis.
- To estimate the importance of individual parameters, VIP scores are calculated, which provide us more information on individual parameters in determining distinct cell fates.

### 2.3 Step 3: Analysis of multistable situation

Due to the complexity of multistability, further analyis is performed as follows.

- Perform PCA over all data, including monostable and multistable data.
- Determine the critical value $V_{c}$ by which the monostable states can be divided into different categories by using only PC1. Otherwise, more PCs need to be incorporated. For example, confidence ellipses are used to divide different cell fates when the first two PCs are used.
- For the data in the multistable date sets, the critical values or the confidence ellipses are used to predict cell fates. Some states are qualitatively different to the monostable states, e.g. the hybrid state E/M state. While other states are qualitatively same to the monostable state, e.g. E or M state.

We next use TS (Gardner *et al.* 2000) and EMT (Tian *et al.* 2013) as illustrative examples to demonstrate the feasibility and efficiency of the methodology. For more details of the approach, see the Supplementary Appendix.

## 3 Results

### 3.1 TS with two random parameters

The switch is consisted of two mutually inhibitory proteins and allows two stable steady states. Both states have a dominant protein expression. The ordinary differential equation (ODE) model with 10 parameters and all basal parameter values are given in the Supplementary Appendix.

**Figure 1.**
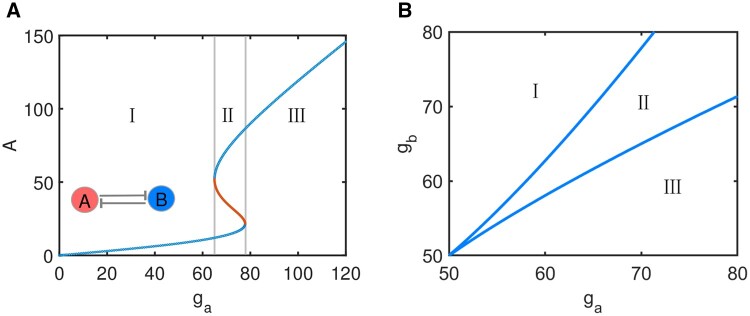
Bifurcation diagrams of the TS. (A) Bifurcation diagram with $g_{a}$ as a control parameter at *g_b_* = 70 nmol l^−1^ h^−1^. (B) Two-parameter bifurcation diagram with $g_{a}$ and $g_{b}$ as control parameters.

To show how the approach is applied, we consider a simple case in which only two parameters $g_{a}$ and $g_{b}$ can be perturbed with the remaining eight parameters fixed at basal values. The bifurcation diagram with $g_{a}$ as a control parameter is shown in Fig. 1A. Based on relevant biological information, the switch has two kinds of steady states: state I (B${}_{\text{high}}$, A${}_{\text{low}}$) and state III (A${}_{\text{high}}$, B${}_{\text{low}}$). When $g_{a}$ and $g_{b}$ are perturbed simultaneously, the bifurcation diagram is shown in Fig. 1B.

Generally, genetic networks operate in noisy cellular environments. When accurate measurements of kinetic parameters are unknown, deterministic bifurcation theory is not suitable yet. To understand dynamical behavior of genetic regulatory networks, parameters are often chosen or perturbed randomly. When $g_{a}$ and $g_{b}$ are randomly chosen, it is assumed that they obey the uniform distribution in the interval [50 80].

**Figure 2.**
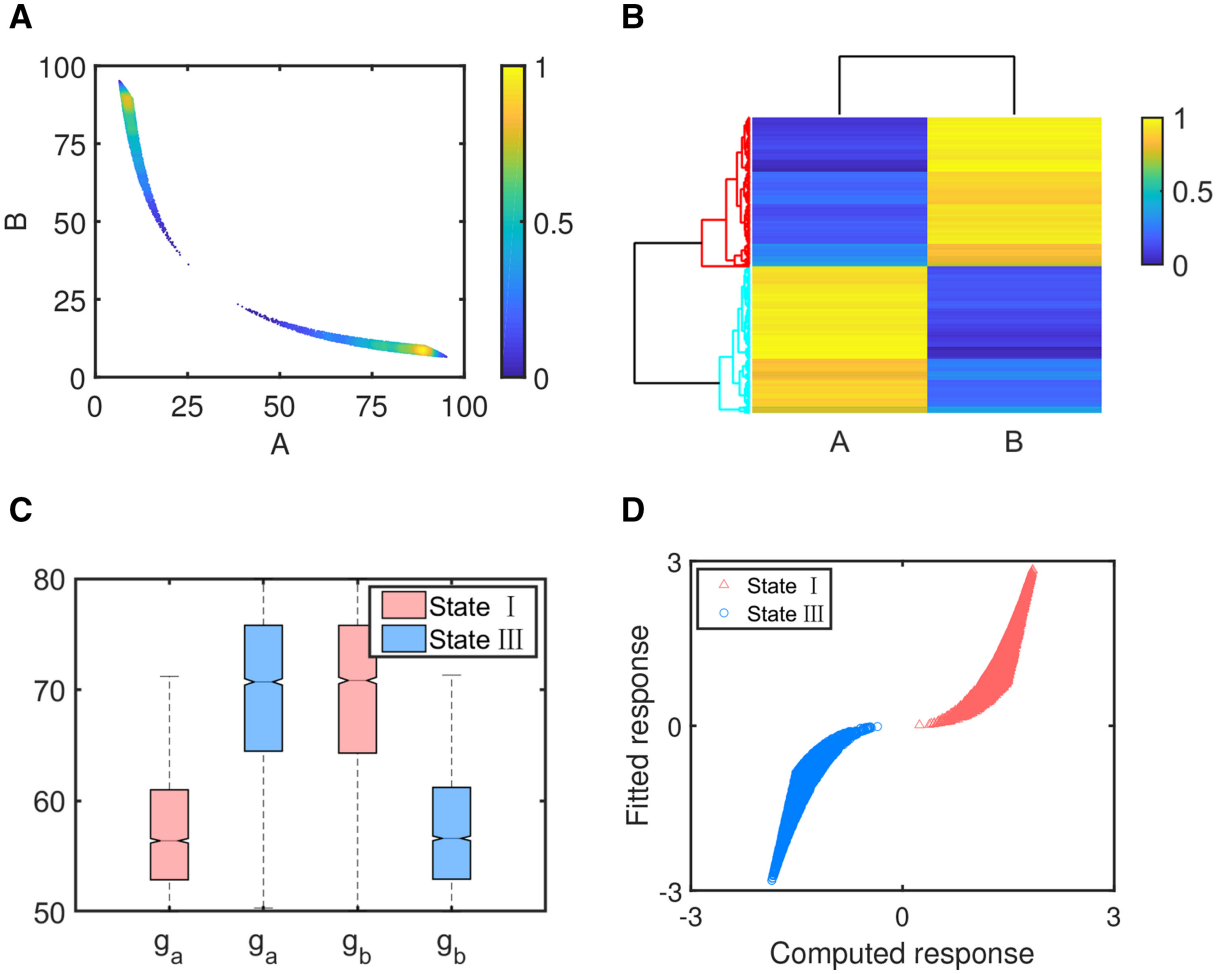
Application of the approach to TS with only randomly chosen $g_{a}$ and $g_{b}$. (A) Scatter plot of steady states of proteins A and B. (B) Average linkage hierarchical clustering analysis of steady states using Euclidean distance. (C) Distributions of $g_{a}$ and $g_{b}$ by box plots. (D) Regression relationship between computed and fitted responses PC1.

Scatter plot of two proteins A and B is shown in Fig. 2A. Despite of large variations in the parameter sets, TS converges quite well into two distinct fates: state I (B${}_{\text{high}}$, A${}_{\text{low}}$) and state III (A${}_{\text{high}}$, B${}_{\text{low}}$), as shown in Fig. 2B. These two fates represent distinct patterns TS can support. Two fates are robust against large perturbation to parameters because the network topology restricts other possible patterns. Generally, various patterns are associated with different cell phenotypes during cellular decision-making processes or fate transition among different states.

Box plot is used to show distributions of the two randomly chosen parameters $g_{a}$ and $g_{b}$ at two distinct states, as shown in Fig. 2C. It is easy to see that at lower $g_{a}$ and higher $g_{b}$, the switch stays at state I. On the contrary, at higher $g_{a}$ and lower $g_{b}$, the switch stays at state III. Due to symmetry network topology of TS, distributions of the two parameters are also symmetry. More importantly, good correspondence between Fig. 1B and Fig. 2C suggests that a good map can be constructed between cell fates and parameters without accurate measurements by the differential distributions.

We further use PLS regression approach, which is an algorithm combining the advantages of three analytical methods: principal component analysis, typical correlation analysis, and multiple linear regression analysis (Geladi and Kowalski 1986, Boulesteix and Strimmer 2007), to predict cell fates under a new combination of $g_{a}$ and $g_{b}$. By clustering analysis, steady states and their corresponding parameters can be divided into two clusters. Then, by PCA, we get the first principal component, i.e. PC1, which accounts for most information contained in the steady state data. Finally, PC1 and the parameter data are used to find the best-fit regression model and obtain regression coefficients. The scatterplot of the estimated PC1 from PCA of the ODE model versus the fitted response though the PLS regression is shown in Fig. 2D.

For the randomly chosen parameters $g_{a}$ and $g_{b}$, the matrix of predictor variables *P* is n×s with s=2 representing the two parameters, and response matrix *X* is n×m with m=2 indicating two proteins A and B. An ensemble of *n*=10 000 parameter sets $(g_{a}$, $g_{b}$) are randomly created and 25% of them allow two coexisting stable steady states (bi-stability), while the others allow only one steady state (mono-stability). We can further make predictions according to the intervals of the fitted response. More exactly, for any parameter set $(g_{a}$, $g_{b}$), when the fitted value is lower than the threshold, i.e. about 0, the TS will stay at state I. Otherwise, it will stay at state III.

Regression analysis is performed to fit and determine regression coefficients. The correlation between PC1 obtained from PCA and fitted data by regression analysis is shown in Fig. 2D. To verify accuracy of classification, 70% of the matrixes *P* and *X* are chosen as training sets, while the remaining 30% are used as test sets (Xu *et al.* 2009). If the classification made by regression analysis is consistent with the results obtained from the ODE model, the classification is true. Otherwise, it is false. Good classification accuracy of the approach corresponding to different confidence intervals (CIs) is shown in Table 1.

**Table 1.** Classification accuracy under two randomly chosen parameters.

CIs	State I (1138 test sets)			State III (1121 test sets)		
	True	False	Accuracy	True	False	Accuracy
90%	1132	6	0.99472759	1121	0	1.0000
95%	1132	6	0.99472759	1121	0	1.0000
99%	1132	6	0.99472759	1121	0	1.0000

### 3.2 TS with all random parameters

**Figure 3.**
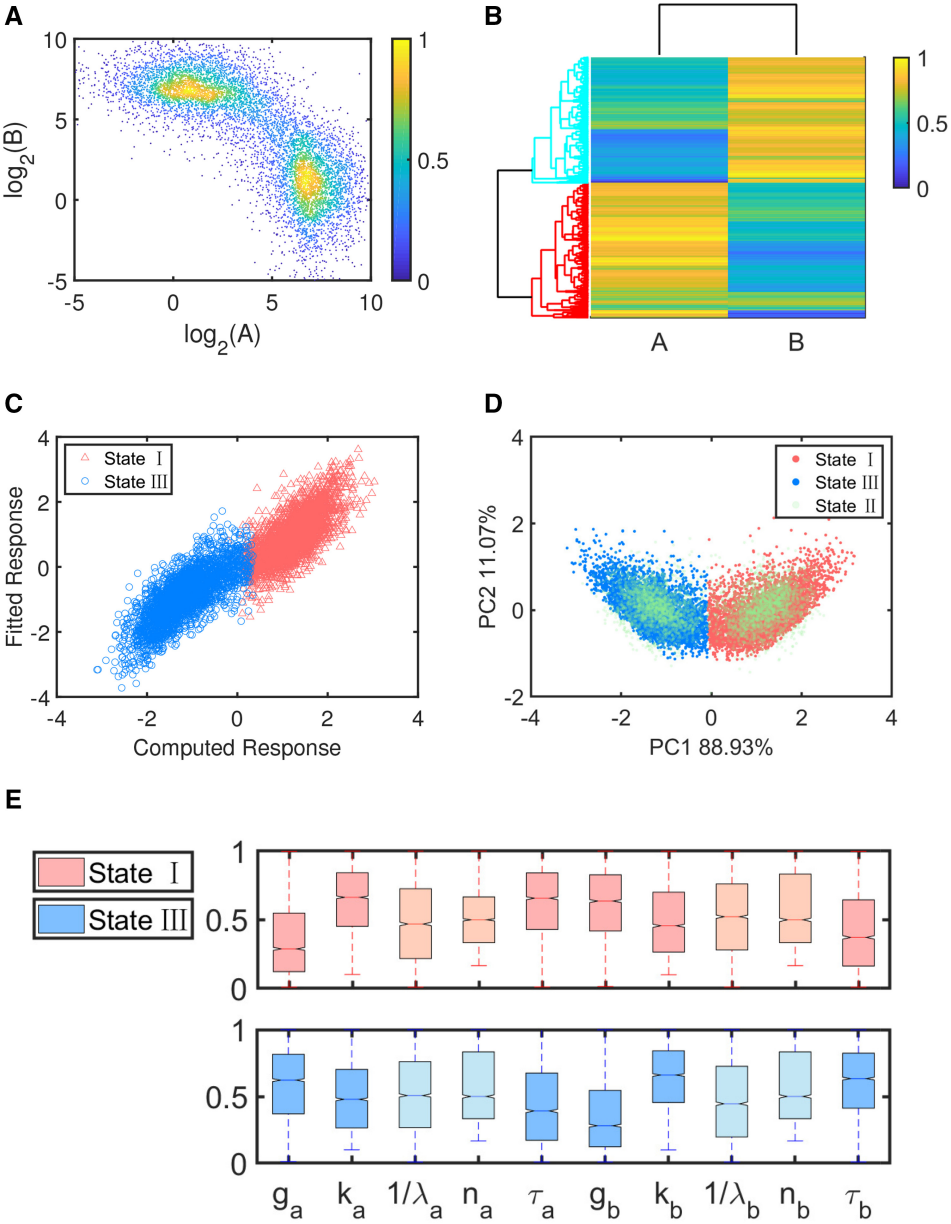
Application of the approach to TS with all randomly varying parameters. (A) Scatter density plot of two proteins. (B) Dendrogram obtained from hierarchically clustering steady state data with average linkage and Euclidean distance. (C) The correlation between the first princiapl component PC1 and the fitted data. (D) Analysis on multistability. (E) Box plots of the 10 randomly varying parameters.

When one or two parameters are perturbed, deterministic bifurcation theory can be applied to analyze state transitions. However, when more parameters are perturbed simultaneously, it becomes difficult to perform bifurcation analysis so as to visualize and understand the roles of different parameters during fate transitions among different states. When all the 10 parameters are randomly perturbed, 10 000 parameter sets are generated. It’s assumed that each parameter obeys the uniform distribution in each corresponding interval, and more details about the intervals can be found in the Supplementary Appendix. For each randomly chosen parameter set, we solve the ODE model and get its steady states. The scatter plot of two protein levels with their distributions and densities is shown in Fig. 3A. Naturally, two qualitatively distinct states with (B${}_{\text{high}}$, A${}_{\text{low}}$) and (A${}_{\text{high}}$, B${}_{\text{low}}$) can be identified. corresponding to state I and III, respectively, as shown in Fig. 3B.

Similarly, differences between PC1 and the fitted data for the monostability case are shown in Fig. 3C. It seems that no large departures from the linear identity mapping although two responses overlap slightly. For the multistability case, PCA over all data is performed, including mono-stable and multistable data, as shown in Fig. 3D. The critical value $V_{c}$ determined according to the known categories of monostable states is about −0.08 by which the category of each state in the multistable datasets (green dots) can be determined. For example, if the PC1 is larger than $V_{c}=-0.08$, the state will be state I. Otherwise, it will be state III.

Box plots are constructed for distributions of ten randomly chosen parameters at two distinct states, as shown in Fig. 3E. Corresponding to normalized parameters, their values are distributed between 0 and 1. Among them, red blocks show the distributions of parameters corresponding to state I, while blue blocks correspond to state III. In addition, dark blocks indicate parameters which are more important. In other words, parameters $g_{a}$, $g_{b}$, $\tau_{a}$, $\tau_{b}$, $k_{a}$, and $k_{b}$ have significant differences at two states and dominate the transition between them. While parameters $\lambda_{a}$, $\lambda_{b}$, $n_{a}$, and $n_{b}$ are evenly distributed at two states. There are no obvious differences between their distributions at two states. Note that for $\lambda_{a}$ and $\lambda_{b}$, there are many outliers. For convenience, we choose $1/\lambda_{a}$ and $1/\lambda_{b}$ in the box plots, instead of $\lambda_{a}$ and $\lambda_{b}$.

Although statistical analysis may tell us the correspondence between steady states and parameters, due to randomization of the parameters, for a specific parameter set, it is generally difficult to predict which stable state a dynamical system will stay at under such a set. To make a prediction, based on the clustering of the steady state data and their corresponding parameter data, we first use PCA to extract the first principal component which describes maximum correlation between response variables. Then, we use the first principal component to perform regression analysis and obtain classification and prediction criterion of explanatory variables, i.e. parameter sets.

We still choose different CIs to check classification accuracy of the approach. It provides good classification accuracy over 85% for CI = 85%, as shown in Table 2. In addition, the overlap between two responses is the main reason why not all judgements are true, as shown in Fig. 3C. Luckily, the overlap interval is not large and we can still identify most of them. Actually, the overlap is not induced by the regression analysis. The overlap occurs mainly because two distinct states get mixed up with each other under certain parameter sets.

**Table 2.** Classification accuracy under all randomly perturbed parameters.

CIs	State I (1115 test sets)			State III (1197 test sets)		
	True	False	Accuracy	True	False	Accuracy
80%	1008	125	0.8421	1091	124	0.8888
85%	1042	159	0.8672	1120	167	0.8502
90%	1070	222	0.8145	1148	229	0.7946

### 3.3 Application to EMT network

**Figure 4.**
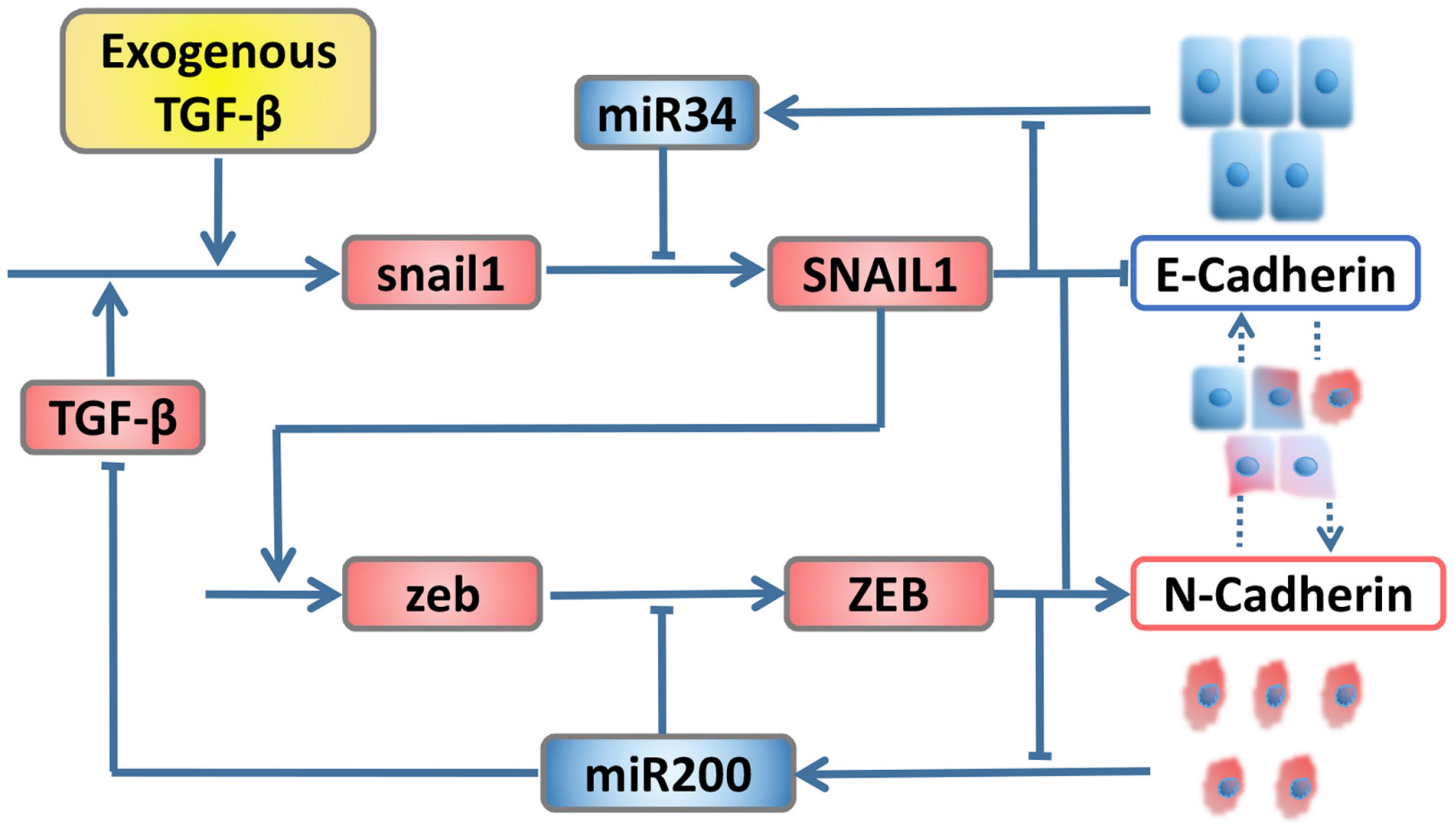
Schematic depiction of core EMT regulatory network.

As an illustrative example, TS is used to show how to construct the map between two cell fates and parameters with accurate measument by regression analysis and differential distribution. Now, we apply it to a more complex EMT network composed of main inducer transforming growth factor TGF-β, cell fate microRNA regulators miR-34 and miR-200, and core transcription factors SNAIL1 and ZEB, as shown in Fig. 4. The model can be described by a set of ODEs with nine state variables and forty-four parameters.

The EMT is the main source of mesenchymal cells involved in tissue repair and the origin of pathological processes. Through this process, properties of epithelial cells change, accompanied with a stronger ability to promote repair and metastasis. Meanwhile, epithelial cells are plastic, and the processes of EMT and MET allow cells to move back and forth between epithelial and mesenchymal states, and during the MET process, mesenchymal cells gradually establish apicobasal polarity through their intrinsic mechanisms.

State transition is essential for the physiological process of inducing pluripotent stem cells from somatic cells. To clearly distinguish three categories of steady states, we divide them into E, E/M, and M types, representing epithelial, hybrid E/M, and mesenchymal cell phenotypes, respectively. To produce each phenotype, the exogenous concentration and production rate of TGF-β are chosen as TGF0∈[0 3] unit and *k_T_* ∈ [0.3 0.5] μM/h, at which the transition is reversible. For each other parameter *p*, it is assumed that it is uniformly distributed in the interval $\left(75\%p_{0},125\%p_{0}\right)$, where $p_{0}$ is the basal value of *p*, as shown in Supplementary Table S1.

**Figure 5.**
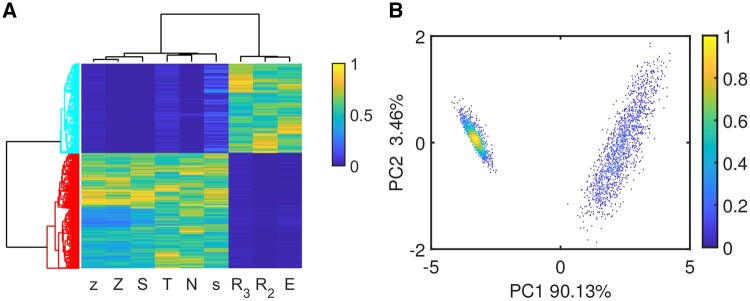
Two qualitatively different clusters, corresponding to epithelial and mesenchymal cells, respectively. (A) Dendrogram obtained from hierarchically clustering the steady state data. (B) The data are projected onto the plane of the first two principal components.

To construct the map, 5000 parameter sets are randomly chosen, at which 2729 parameter sets produce only E or M state by computing the ODE model of nine state variables, namely “T,” “s,” “S,” “R3,” “z,” “Z,” “R2,” “E,” and “N,” representing the levels of endogenous TGF-β, snail, SNAIL, miR-34, zeb, ZEB, miR-200, E-cadherin, and N-cadherin, respectively. By the average linkage unsupervised hierarchical clustering analysis through the Euclidean distance metric method, the clusters form a tree-type structure based on the hierarchy, as shown in Fig. 5A. Two qualitatively different clusters are obtained, corresponding to epithelial and mesenchymal cells, respectively. The scatterplot of two steady state clusters against the first two principal components, i.e. PC1 and PC2, is shown in Fig. 5B. We can intuitively regard that there are two types of phenotypes. They are distributed over both wings and are dense and clearly divisible. Note that to improve distinguishability of two distinct phenotypes, we remove the data of hybrid E/M state and the data of the E and M phenotypes under the same parameter combinations at which the E/M phenotype exists. After clustering and PCA analysis, distribution of these data is shown in Fig. 5B.

**Figure 6.**
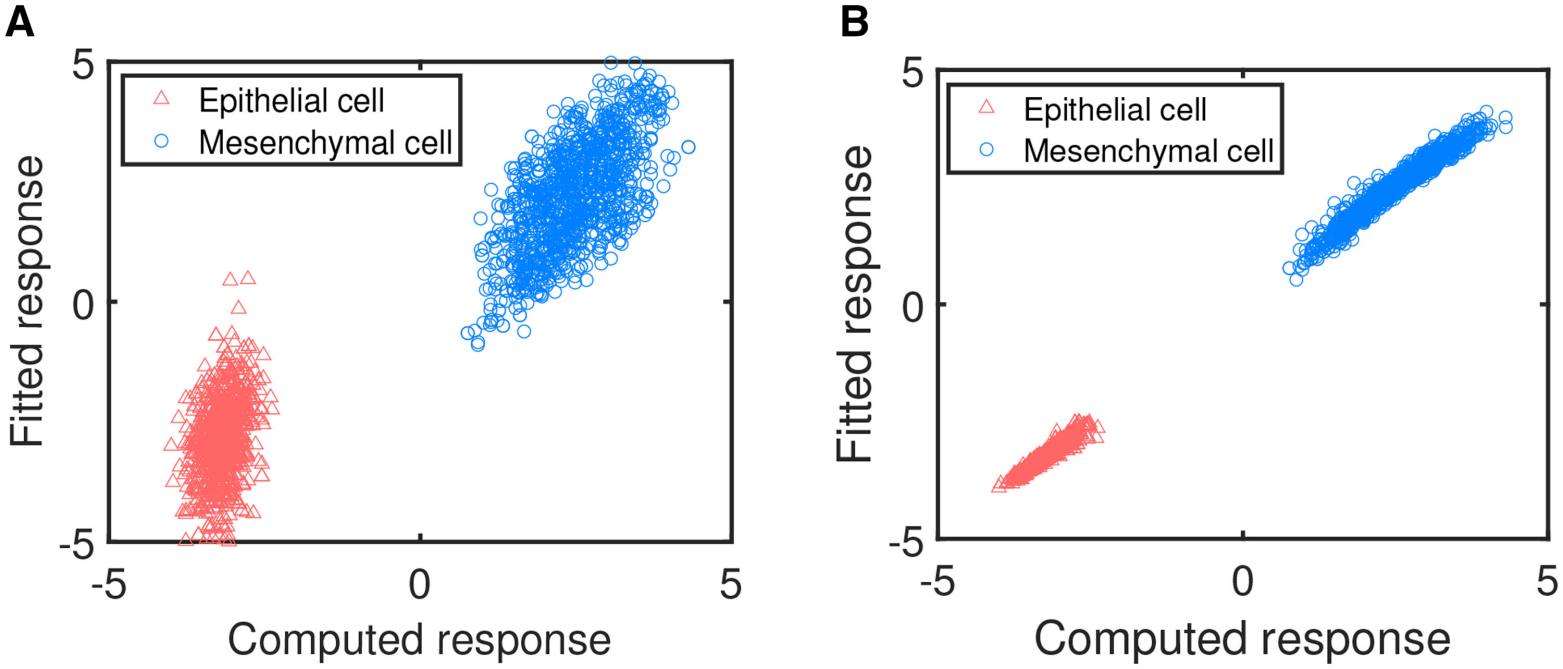
The correlation between the first principal component PC1 and the fitted response. (A) Two distinct steady state sets of E and M are integrated when fitting. (B) Better fitting when regression is performed for each phenotype separately.

To quantify the relationship between the explanatory variables, i.e. parameter sets, and the response variables, i.e. steady state sets, we use PLS regression to predict which state the system will stay at under a new parameter set. Now we fit the regression model using parameter set as the explanatory variables and PC1 of the steady state sets as the response variable, which contains 90.13% information revealed by the stable steady state data. The scatter plot of response obtained from the first principal component, i.e. PC1, of the ODE model versus the fitted response though PLS regression analysis is shown in Fig. 6. After regression model is fitted, we can use it as classification or prediction criterion. Good classification accuracy is obtained, as shown in Table 3.

**Table 3.** Classification accuracy of EMT network.

CIs	Stage E (358 test sets)			Stage M (462 test Sets)		
	True	False	Accuracy	True	False	Accuracy
90%	343	0	0.9581	438	0	0.9481
95%	352	1	0.9832	454	1	0.9827
99%	356	2	0.9944	460	3	0.9935

The classification accuracy is estimated as follows. We take 70% of the steady state data and their corresponding parameter data as the training sets, and the remaining 30% data as the test sets. More exactly, we use the sets of 44 parameters as the explanatory variables and the steady state sets of nine state variables as the response variables. We choose the first principal component PC1 which contains most of the information on the steady states of the nine variables. Table 3 shows good classification accuracy. For example, when the CI 99% or 95% is chosen, classification accuracy exceeds 98%. In other words, more than 98% correct one-to-one correspondence between a parameter set and its corresponding epithelial or mesenchymal phenotype can be predicted. For any parameter set, when the fitted response value is lower than −1.1594, the phenotype will be judged as epithelial, while when the value is higher than 1.658 $\times 10^{-4}$, the phenotype will be judged as mesenchymal, as shown in Fig. 6A.

When two distinct steady state sets of E and M are integrated to fit the regression model, although good classification accuracy can be obtained, the fitting effect is not so precise because the unique regression model needs to fit two distinct steady state sets at the same time, which results in the existence of deviation from the identity map, as shown in Fig. 6A. To further obtain the ranking of all parameters according to their importance in determining cell fates, when cell fate is predetermined, we perform regression for each phenotype separately and obtain better fitting results, as shown in Fig. 6B. In other words, we get two more precise regression models, fitting two distinct steady state sets of E and M.

**Figure 7.**
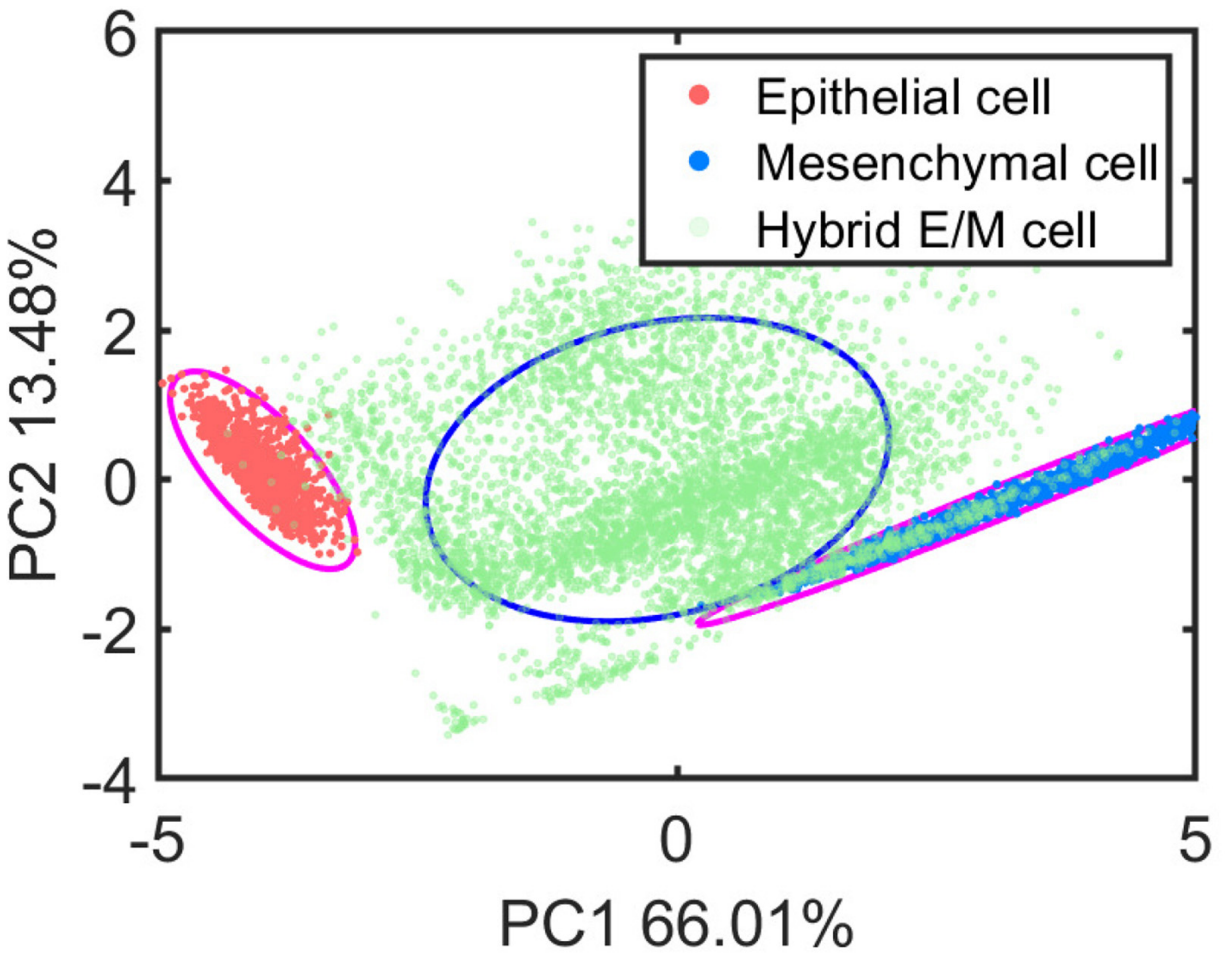
Classification of the multiple stable states in the EMT network by confidence ellipse with the first two PCs.

For the tristable case of EMT network, the scatterplot on PC1-PC2 plane is shown in Fig. 7. When the data in multistable datasets are distributed in the same ellipse region with one of monostable states, the cell fate in the same ellipse are thought as the same. For the multiple case, all stable steady states under a given parameter set are represented in green color although they may belong to different cell types. The classification of E and M states have been performed by the fitting analysis. Therefore, some data are considered as E (green dots in the ellipse of E cells) or M (green dots in the ellipse of M cells) state. While others are considered as hybrid state, as shown by the green dots in the blue ellipse. The number of clusters is determined by relevant biological information and steady state analysis. More details on how to define the confidence ellipse can be found in the Supplementary Appendix.

It is worth noting that complexity of multistability may induce a biased estimate when one or more steady states are missed and incorrectly classified as monostability. The rate at which this happens may depend on various factors, such as the size of the region of attraction of stable steady states, the sampling distribution, and the number of sampled initial conditions.

**Figure 8.**
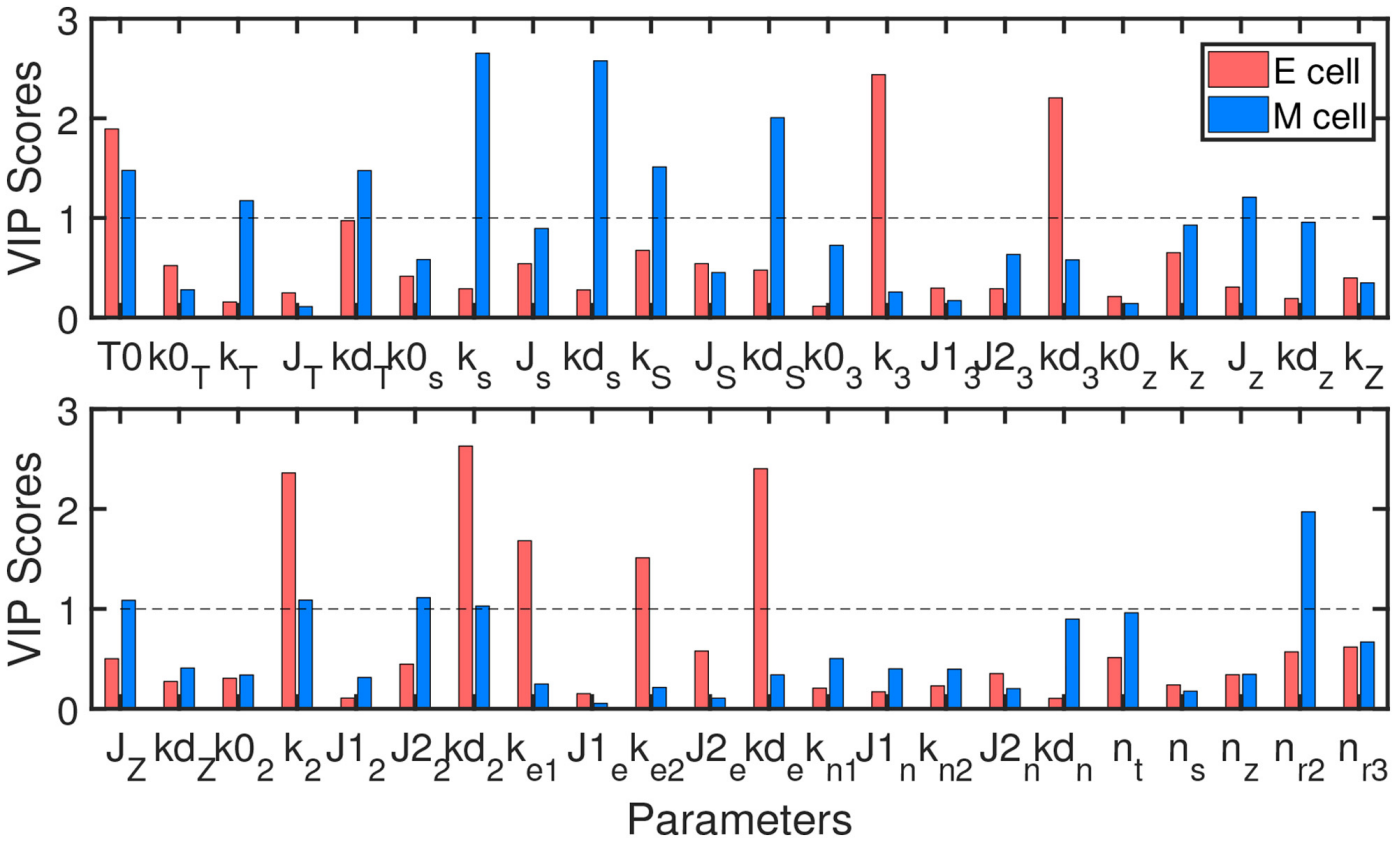
VIP score of each parameter in EMT network. The dotted lines indicate the threshold of VIP scores.

Using the better regression models corrsponding to Fig. 6B, we can understand importance of each parameter in determining each cell fate. Relative importance of each parameter in EMT network is shown in Fig. 8. Red and blue bars depict VIP score of each parameter, which represents importance ordering of each parameter in determining epithelial and mesenchymal fates, respectively. For example, it is known that TGF-β is an effective driver of EMT, and fate transition is sensitive to exogenous concentration of TGF-β. Therefore, exogenous TGF-β treatment, i.e. the parameter *T*0, is important in both E and M states.

In addition, when fitting to epithelial cells, the production rates of miR-34 and miR-200, i.e. $k_{2}$ and $k_{3}$, and the production rates of E-cadherin, i.e. $k_{e1}$ and $k_{e2}$ are clearly larger than the threshold 1. Their degradation rates, i.e. $kd_{2}$, $kd_{3}$, and $kd_{e}$, are also important, but in the opposite direction. Similarly, mesenchymal cells are normally controlled by high TGF-β, ZEB, and SNAIL1 levels. Their production rates, degradation rates, and translation rates are important, in either positive or negative direction.

**Figure 9.**
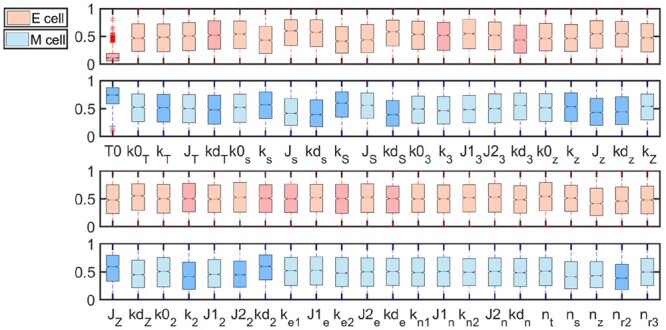
Box plots of all randomly chosen parameters in EMT network.

Existing studies show that miR-34 and miR-200 are highly expressed in epithelial cells, while TGF-β, SNAIL1, and ZEB are highly expressed in mesenchymal cells. Consistent with these evidences, box plots show the same tendency. After taking absolute value of the PLS regression coefficients and sorting them, their importance ordering can be extracted. It is known that TGF-β, SNAIL1, and ZEB are highly expressed in mesenchymal cells. Correspondingly, values of parameters *T*0, $k_{s}$, $k_{S}$, and $kd_{3}$ are obviously larger, as shown in Fig. 9, which results in higher SNAIL1 and ZEB levels. While larger $kd_{3}$ induces fast degradation of miR-34, and therefore low miR-34 level. Similarly, the negatively correlated parameters $J_{s}$, $kd_{s}$, $kd_{S}$, and $kd_{z}$ with apparently smaller values can induce the same tendency, i.e. higher TGF-β, SNAIL1, and ZEB levels, as shown in Fig. 9.

The consistency between Fig. 8 and Fig. 9 indicates the importance ranking of all parameters is reasonable. The statistical analysis shown in Fig. 9 tell us systematic perturbation information so we can perform systematical perturbation simultaneously so as to realize or control cell fate transition more easily. While the ranking analysis shown in Fig. 8 provides us more intuitive information on individual perturbation than the differential distributions of systematical perturbation. In short, the approach integrating systematical perturbation, clustering analysis, PCA, and fitting analysis can help us to analyze dynamics of regulatory networks, make predictions on which state a dynamical system can stay at under a specified parameter combination, and control and realize cell fate transitions among different states. The insight gained from this study is expected to provide a basis for constructing maps between cell fates and parameters without accurate measurement by systematic perturbation. In addition, the approach presented here can be expected to analyze other biological networks related to cell fate decisions and transitions.

## 4 Conclusion

Cell fate decisions and transitions play critical roles in almost every cellular process, and abnormal changes induced by various perturbations may result in developmental disorders and diseases such as cancers. Accumulating evidences indicate that interplay of multiple regulators such as activators and repressors determines almost all developmental events and cell fate decisions. Therefore, systematic perturbation analysis is becoming more and more important to identify key regulators or their combinations. There have been extensive studies on systematic perturbations, but maps between cell fates and parametric conditions are still less clear and need to be further investigated because the relations between steady states and kinetic parameters are usually nonlinear and complex. Up to now, there is no general theory on how to infer relations between steady states and parametric conditions.

Computational and experimental studies have identified an abundance of such maps, e.g. EMT and ICM. In this paper, we present a general computational approach for constructing maps between cell fates and randomized parameters by systematic perturbations. The approach integrates systematical perturbations, clustering analysis, PCA, and fitting analysis. In agreement with experimental observations, the approach can account for some functional features of certain parameter combinations in cell fate decisions such as EMT. In addition to PCA, other approaches such as the integrated approach by combining SVM and parallel-coordinates plots can also result in a tremendously reduced computational complexity (Hasenauer *et al.* 2012).

For a long time, we were limited to studying steady states by bifurcation analysis under perturbation to one or two parameters. When more parameters are perturbed simultaneously, it becomes difficult to perform bifurcation analysis so as to visualize and understand the roles of certain parameter combinations during fate transitions among different states. Systematical perturbations provide more insight into valuable information contained in certain parameter combinations. While importance of each parameter in determining each cell fate can be further assessed by estimating VIP scores.

Although the relations between steady states and kinetic parameters are usually nonlinear, we still use linear regression to classify and predict which state a dynamical system will stay at under certain parametric conditions. When applied to TS, ICM, and EMT, good prediction accuracy of the approach indicates its good effectiveness in revealing dynamics of genetic networks. It is worth noting that compared with bistability, multistability is generally more complex. Therefore, when performing classification, only the first PC1 is always not enough, and more principal components are required. The approach presented here can be applied to analyze other biological networks related to cell fate decisions and transitions and further to construct maps between cell fates and certain parametric conditions. In addition, the approach is especially helpful in understanding crucial roles of certain parameter combinations during fate transitions among different states.

## Supplementary Material

btad624_Supplementary_Data
