# Supplementary material for: Constructing maps between distinct cell fates and parametric conditions by systematic perturbations
Source: Bioinformatics. 2023 Oct 13;39(10):btad624. doi: 10.1093/bioinformatics/btad624 (PMC10603594; doi:10.1093/bioinformatics/btad624)
Supplement: btad624_Supplementary_Data [file btad624_supplementary_data.pdf]

Appendix

Ruoyu Tang, Xinyu He, Ruiqi Wang*

June 18, 2023

1 TS

The TS model with ten parameters takes the form

$$\dot{A} = g_a H^-(B) - k_a A, \tag{1}$$

$$\dot{B} = g_b H^-(A) - k_b B. \tag{2}$$

Unless otherwise noted, all parameters obey uniform distributions [1].

In the ODEs, g_a represents the production rate of gene A and belongs to [1,100], and k_a represents the degradation rate of gene A and the interval is $[0.1, 1]$. $H^-(B)$ denotes inhibition rate of A by B, and is given by

$$H^-(B) = \lambda_b + \frac{1-\lambda_b}{1+\left(\frac{B}{\tau_b}\right)^{n_b}},$$

where τ_b is the threshold of B and follows the half-function rule; n_b is the integer Hill coefficient selected from $[1, 6]$. λ_b is the fold change with $1/\lambda_b \in [1, 100]$.

2 EMT network

EMT can be described by the following set of ODEs with nine state variables and forty-four parameters [2].

$$\frac{d[T]}{dt} = k0_T + \frac{k_T}{\left(1 + \frac{[R2]}{J_T}\right)^{n_{r2}}} - kd_T[T], \tag{3}$$

$$\frac{d[s]}{dt} = k0_s + k_s \frac{\left(\frac{[T]+T0}{J_s}\right)^{n_t}}{1 + \left(\frac{[T]+T0}{J_s}\right)^{n_t}} - kd_s[s], \tag{4}$$

$$\frac{d[S]}{dt} = k_S[s] \frac{1}{1 + \left(\frac{[R3]}{J_S}\right)^{n_{r3}}} - kd_S[S], \tag{5}$$

$$\frac{d[R3]}{dt} = k0_3 + k_3 \frac{1}{1 + \left(\frac{[S]}{J1_3}\right)^{n_s} + \left(\frac{[Z]}{J2_3}\right)^{n_z}} - kd_3[R3], \tag{6}$$

$$\frac{d[z]}{dt} = k0_z + k_z \frac{\left(\frac{[S]}{J_z}\right)^{n_s}}{1 + \left(\frac{[S]}{J_z}\right)^{n_s}} - kd_z[z], \tag{7}$$

$$\frac{d[Z]}{dt} = k_Z[z] \frac{1}{1 + \left(\frac{[R2]}{J_Z}\right)^{n_{r2}}} - kd_Z[Z], \tag{8}$$

$$\frac{d[R2]}{dt} = k0_2 + k_2 \frac{1}{1 + \left(\frac{[S]}{J1_2}\right)^{n_s} + \left(\frac{[Z]}{J2_2}\right)^{n_Z}} - kd_2[R2], \tag{9}$$

$$\frac{d[E]}{dt} = k_{e1} \frac{1}{1 + \left(\frac{[S]}{J1_e}\right)^{n_s}} + k_{e2} \frac{1}{1 + \left(\frac{[Z]}{J2_e}\right)^{n_z}} - kd_e[E], \tag{10}$$

$$\frac{d[N]}{dt} = k_{n1} \frac{\left(\frac{[S]}{J1_n}\right)^{n_s}}{1 + \left(\frac{[S]}{J1_n}\right)^{n_s}} + k_{n2} \frac{\left(\frac{[Z]}{J2_n}\right)^{n_z}}{1 + \left(\frac{[Z]}{J2_n}\right)^{n_z}} - kd_n[N]. \tag{11}$$

S1 Table. The basal parameter values. Except for exogenous concentration of TGF-β, i.e., T0, parameters are uniformly distributed in $(75\%p_0, 125\%p_0)$, where p_0 is the basal value of each parameter.

Parameters	Value	Unit	Description
$T0$	0-3	unit	Exogenous concentration of TGF-β
$k0_T$	0.06	$\mu M/hr$	Basal production rate of TGF-β
k_T	0.4	$\mu M/hr$	Production rate of TGF-β
J_T	0.06	$\mu M/hr$	Michaelis constant of miR200-dependent inhibition of TGF-β expression
kd_T	0.6	$/hr$	Degradation rate of TGF-β
$k0_s$	0.0006	$\mu M/hr$	Basal transcription rate of snail1
k_s	0.03	$\mu M/hr$	Transcription rate of snail1
J_s	1.6	μM	Michaelis constant of TGF-β-dependent snail1 translation
kd_s	0.09	$/hr$	Degradation rate of snail1 mRNA
k_S	17	$\mu M/hr$	Translation rate of snail1 mRNA
J_S	0.08	μM	Michaelis constant of miR34-dependent inhibition of snail1 translation
kd_S	1.66	$/hr$	Degradation rate of SNAIL1
$k0_3$	0.0012	$\mu M/hr$	Basal production rate of miR-34
k_3	0.012	$\mu N/hr$	Production rate of miR-34
$J1_3$	0.15	μM	Michaelis constant of SNAIL1-dependent inhibition of miR-34 production
$J2_3$	0.36	μM	Michaelis constant of ZEB-dependent inhibition of miR-34 production
kd_3	0.035	$/hr$	Degradation rate of miR-34
$k0_z$	0.003	$\mu M/hr$	Basal transcription rate of zeb
k_z	0.06	$\mu M/hr$	Transcription rate of zeb
J_z	3.5	μM	Michaelis constant of SNAIL1-dependent zeb transcription
kd_z	0.09	$/hr$	Degradation rate of zeb mRNA
k_Z	17	$\mu M/hr$	Translation rate of zeb mRNA
J_Z	0.06	μM	Michaelis constant of miR34-dependent inhibition of zeb mRNA translation
kd_Z	1.66	$/hr$	Degradation rate of ZEB
$k0_2$	0.0002	$\mu M/hr$	Basal production rate of miR-200
k_2	0.012	$\mu M/hr$	Production rate of miR-200
$J1_2$	5	μM	Michaelis constant of SNAIL1-dependent inhibition of miR-200 production
$J2_2$	0.2	μM	Michaelis constant of ZEB-dependent inhibition of miR-200 production
kd_2	0.035	$/hr$	Degradation rate of miR-200
k_{e1}	1	$\mu M/hr$	Production rate 1 of E-cadherin production
$J1_e$	0.2	μM	Michaelis constant of SNAIL1-dependent inhibition of E-cadherin production
k_{e2}	0.6	$\mu M/hr$	Production rate 2 of E-cadherin production
$J2_e$	0.5	μM	Michaelis constant of ZEB-dependent inhibition of E-cadherin production
kd_e	0.5	$/hr$	Degradation rate of E-cadherin
k_{n1}	1	$\mu M/hr$	Production rate 1 of N-cadherin production
$J1_n$	0.2	μM	Michaelis constant of SNAIL1-dependent N-cadherin production
k_{n2}	0.6	$\mu M/hr$	Production rate 2 of N-cadherin production
$J2_n$	0.5	μM	Michaelis constant of ZEB-dependent N-cadherin production
kd_n	0.5	$/hr$	Degradation rate of N-cadherin
n_t	2	-	Hill coefficient of TGF-β-dependent SNAIL1 expression
n_s	2	-	Hill coefficient of SNAIL1-dependent activation or inhibition
n_z	2	-	Hill coefficient of ZEB-dependent inhibition
n_{r2}	2	-	Hill coefficient of miR-200-dependent inhibition
n_{r3}	2	-	Hill coefficient of miR-34-dependent inhibition

3 Application to inner cell mass (ICM)

In order to better demonstrate wide application of the method, we apply it to study cell fate decisions in the inner cell mass composed of two antagonistic transcription factors, i.e., Nanog and Gata6, which control the differentiation of the ICM into Epi and PrE. See [3] for more details on the model. The network composed of Nanog, Gata6, Fgfr2 (FR), Fgfr/Erk (ERK), and the averaged level of Fgf4 secreted by the cell and its four neighbors (Fs), is shown in Fig. S1.

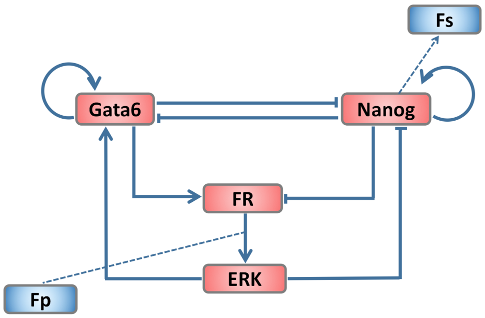

Figure 1: **The regulatory network controlling the differentiation of the inner cell mass into Epi and PrE phenotypes.**

The Fgf/RTK signaling pathway, Nanog and Gata6 are crucial factors in balancing cell fate specification between Epi and PrE. Through the influence of their Fgf/RTK environment, ICM cells will present a larger proportion of cells differentiating into PrE by culturing with recombinant Fgf4 [4], while inhibiting the Erk signaling pathway also prevents their differentiation into PrE and maintains in a pluripotent state [5].

To clearly distinguish three categories of steady states, we divide them into Epi-like, ICM-like, and PrE-like types, representing different cell phenotypes, respectively. To generate each phenotype, the extracellular concentration of Fgf4 is chosen as Fp $\in$ [0 0.15] unit and other parameters are uniformly distributed in the interval $(75\%p_0, 125\%p_0)$, where p_0 is the basal value of p, as shown in Table. S4.

To construct the map, 10,000 parameter sets are randomly chosen, at which 5,417 parameter sets produce only Epi-like or PrE-like state by computing the ODE model of five state variables, namely 'G', 'N', 'FR', 'ERK', and 'Fs', representing the levels of Gata6, Nanog, Fgfr2, Erk, and the amount of Fgf4 proteins secreted, respectively.

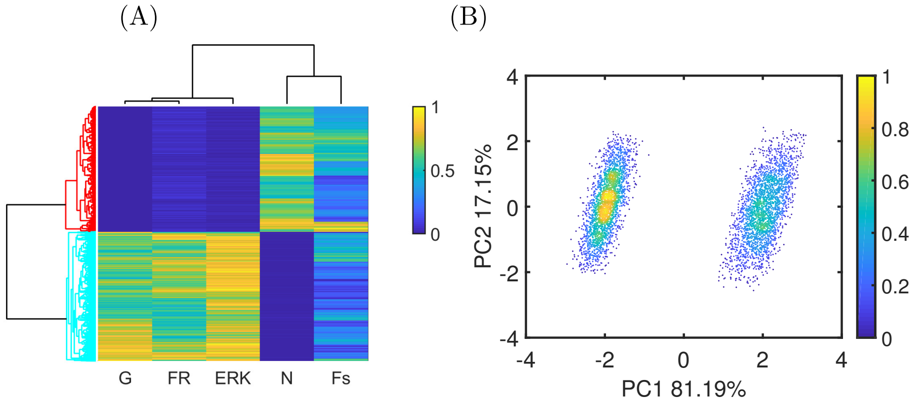

Figure 2: **Two qualitatively different clusters, corresponding to Epi-like and PrE-like cells, respectively.** (A) Dendrogram obtained from hierarchically clustering the steady state data. (B) The data are projected onto the plane of the first two principal components.

By the average linkage unsupervised hierarchical clustering analysis through the Euclidean distance metric method, the clusters form a tree-type structure based on the hierarchy, as shown in Fig. S2(A). Two qualitatively different clusters are obtained, corresponding to Epi-like and PrE-like state, respectively. The scatterplot of two steady state clusters against the first two principal components, i.e., PC1 and PC2, is shown in Fig. S2B. Note that to improve distinguishability of two distinct phenotypes, we remove the data of coexistence of bistable or tri-stable under the same parameter combinations.

Then, we use PLS regression to predict which state the system will stay at under a new parameter set. Now we fits the regression model using parameter sets and PC1 of the steady state sets as before, which contains 81.19% information revealed by the stable steady state data. The fitted response though PLS regression analysis is shown in Fig. 3. Classification results show good accuracy, and more than 98% correct one-to-one correspondence between a parameter set and its corresponding Epi-like or PrE-like phenotype can be predicted, as shown in Table 2. For any parameter set, when the fitted response value is lower than -0.00074, the phenotype will be judged as Epi-like. While when the value is higher than 0.4738, the phenotype will be judged as PrE-like, as shown in Fig. 3A. When cell fate is pre-determined, we perform regression for each phenotype separately and obtain better fitting results and importance order, as shown in Fig. 3B.

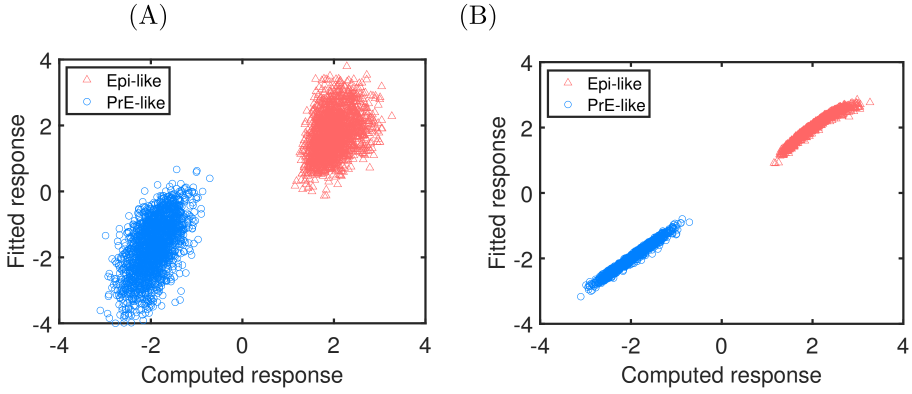

Figure 3: **The correlation between the first principle component PC1 and the fitted response.** (A) Two distinct steady state sets of Epi-like and PrE-like are integrated when fitting. (B) Better fitting when regression is performed for each phenotype separately.

For the tri-stable case, we also use elliptical regions for classification, as shown in Fig. S4A. Some data distributed in the same ellipse as Epi and PrE cells are considered as Epi-like (green dots in the ellipse of Epi-like region) or

Table 2: **Classification accuracy of ICM network**

CIs	Epi -like (780 Test sets)			PrE-like (807 Test sets)		
	True	False	Accuracy	True	False	Accuracy
90%	747	0	0.9577	778	4	0.9641
95%	766	1	0.9821	792	4	0.9814
99%	779	18	0.9769	803	14	0.9827

PrE-like (green dots in the ellipse of PrE-like region) state. While others are considered as ICM-like state, as shown by the green dots in the blue ellipse.

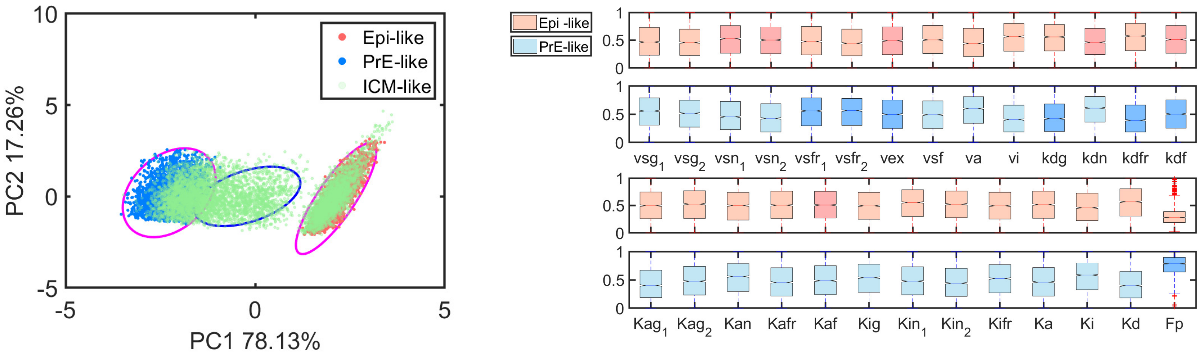

Figure 4: **Steady states and parameters distribution of different phenotypes.** (A) Classification of the multiple stable states in the ICM network by confidence ellipses with the first two PCs. (B) Box plots of all parameters.

Finally, we use boxplots to represent the differences in values of random parameters at different phenotypes and mark out the important ones, as shown in Fig. S4 B, which corresponds well to biological facts. For example, existing studies show that Nanog is necessary for the differentiation of Epi cells, whereas Gata6 is required for producing the PrE epithelium in vitro and in vivo [3]. Moreover, the activation or the blockade of the Fgf/RTK pathway biases cell fate specification towards either PrE or Epi, respectively. Consistent with these evidences, box plots show the same tendency. More exactly, values of parameters vsn_1 and vsn_2 are obviously larger in Epi cells, which results in higher Nanog level. While the negatively correlated parameter kdn with apparently smaller values can induce the same tendency. Similarly, larger $vsfr_2$ and smaller kdg induce high expression of Gata6 in PrE cells. Fp, i.e., the extracellular concentration of Fgf4, is important in both cells. It is significantly low expressed in Epi and significantly high in PrE cells.

4 Parameters related to ICM

Table 3: Except for extracellular concentration of Fgf4 , i.e., Fp, parameters are uniformly distributed in $(75\%p_0, 125\%p_0)$, where p_0 is the basal value of each parameter.

Parameters	Basal values	Description
Fp	0-0.15	Extracellular concentration of Fgf4
vsg_1	1.022	Maximum rate of Gata6 synthesis caused by ERK activation
vsg_2	0.85	Maximum rate of Gata6 synthesis caused by its auto-activation
vsn_1	0.856	Basal rate of Nanog synthesis
vsn_2	1	Maximum rate of Nanog synthesis caused by its auto-activation
$vsfr_1$	2.8	Basal rate of Fgfr2 synthesis
$vsfr_2$	2.8	Maximum rate of Fgfr2 synthesis caused by Gata6 activation
vex	0.1	Basal rate of Fgf4 synthesis
vsf	0.6	Maximum rate of Fgf4 synthesis caused by Nanog activation
va	20	ERK activation rate
vi	3.3	ERK inactivation rate
kdg	1	Gata6 degradation rate
kdn	1	Nanog degradation rate
$kdfr$	1	Fgfr2 degradation rate
kdf	0.077	Fgf4 degradation rate
Kag_1	0.28	Threshold constant for the activation of Gata6 synthesis by ERK
Kag_2	0.55	Threshold constant for Gata6 auto-activation
Kan	0.55	Threshold constant for Nanog auto-activation
$Kafr$	0.5	Threshold constant for the activation of Fgfr2 synthesis by Gata6
Kaf	5	Threshold constant for the activation of Fgf4 synthesis by Nanog
Kig	2	Threshold constant for the inhibition of Gata6 synthesis by Nanog
Kin_1	0.28	Threshold constant for the inhibition of Nanog synthesis by ERK
Kin_2	2	Threshold constant for the inhibition of Nanog synthesis by Gata6
$Kifr$	0.5	Threshold constant for the inhibition of Fgfr2 synthesis by Nanog
Ka	0.7	Michaelis constant for activation of the ERK pathway
Ki	0.7	Michaelis constant for inactivation of the ERK pathway
Kd	2	Michaelis constant for activation of the ERK pathway by Fgf4
r	3	Hill coefficient for the activation of Gata6 synthesis by ERK
s	4	Hill coefficient for Gata6 auto- activation
q	4	Hill coefficient for the inhibition of Gata6 synthesis by Nanog
u	3	Hill coefficient for the inhibition of Nanog synthesis by ERK
v	4	Hill coefficient for Nanog auto-activation
w	4	Hill coefficient for the inhibition of Nanog synthesis by Gata6
x	1	Hill coefficient for the inhibition of Fgfr2 synthesis by Nanog
y	1	Hill coefficient for the activation of Fgfr2 synthesis by Gata6
z	4	Hill coefficient for the activation of Fgf4 synthesis by Nanog

5 Main method

The approach is developed with the focus mainly on how to construct maps between distinct cell fates and parametric conditions by systematic perturbations.

It starts by randomly perturbing all parameters based on basal values within a defined interval to generate steady state data, which are then standardized and normalized for next operation.

5.1 Data preprocessing

For each randomly perturbed parameter, we obtained the steady states. Because of their different units or orders of magnitude, each parameter needs standardization and each steady state should be normalized too before systematic analysis.

Standardization:

$$\frac{p_i - min(p_i)}{max(p_i) - min(p_i)} \to p_i.$$

Normalization:

$$\frac{x_i - \mu(x)}{\sigma(x)} \to x_i.$$

Here $min(p_i)$, $max(p_i)$, $\mu(x)$, and $\sigma(x)$ denote the minimum and maximum of p_i, mean and standard deviation of x, respectively.

5.2 Identify the multi-stability

For each parameter set $p = (p_1, p_2, \cdots, p_s)^T \in \mathbb{R}^s$, one or more solutions can be obtained by solving the ODEs, i.e. monostability or multistability. We use it as the class label of training sets and then perform supervised kernel support vector machines (KSVM) classification by running holdout cross-validation. The test accuracy is around 98.6% and 87.6% for TS and EMT network, respectively. For each parameter set chosen within the perturbation intervals, we can predict whether it is monostable or multistable under the given parameter set.

5.3 Clustering

For the case of monostability, we use clustering to identify distinct robust state categories. Here we applied average linkage unsupervised hierarchical clustering analysis through the Euclidean distance metric method, as shown in Fig 2B, Fig 3B and Fig 5A. Each column represents a state variable, i.e., a molecule, x_i $(i = 1, \cdots, m)$, and each row represents a stable steady state $x = (x_1, x_2, \cdots, x_m)^T \in \mathbb{R}^m$ of the dynamical system at a given parameter set $p = (p_1, p_2, \cdots, p_s)^T \in \mathbb{R}^s$.

5.4 Dimensionality reduction

Steady state data are generally high-dimensional, and therefore hard to interpret intuitively. Dimensionality reduction needs to be performed by PCA for linearly divisible case or Kernel Principal Component Analysis (KPCA) for linearly indivisible case, which essentially seeks the derived coordinate to capture as much variability in the data as possible.

5.5 Divide the dataset

To verify the classification performance of the approach, for all clusters, 70% of the steady state sets, i.e., $X_{q' \times m}$, and parameter sets related to $X_{q' \times m}$, i.e., $P_{q' \times s}$, form the training sets. Relatively, the remaining 30% act as the test sets.

Through the learning of training sets by PLS regression, we obtain the coefficients and substitute them into test sets. The theoretical details can be understood in the following explanations. The obtained results are compared with known clusters to validate classification efficiency of the method.

6 Principle of PLS regression and VIP score

To construct maps between cell fates and their perturbation mechanisms, many regression methods can be considered [6]. Different from other linear models, such as least squares method, PLS regression is helpful to deal with the noisy and multicollinearity relationship. Compared with nonlinear models, PLS regression hyperplane is a multivariable linear equation, and its regression coefficients can visually indicate the importance ordering of parameters. Besides, PLS regression has stronger interpretability than machine learning methods, and the latter is more of a "black box" model, or more annoying computational complexity. Additionally, PLS regression deals with the multidimensional explanatory variables by extracting latent variables, which is fully consistent with the research related to biological networks.

Based on the actual characteristics of biological networks, the strength of simple PLS regression calculation, strong interpretability and relatively high prediction accuracy, we choose PLS regression to construct maps between cell fates and certain parametric conditions.

For the given explanatory variables $P_{q'\times s}$ and monostable steady state data $X_{q'\times m}$, we first extract the first pair of vectors of them and call them t_1 and u_1, which satisfy the following equations [7, 8]

$$t_1 = Pw_1 \quad \text{with} \quad \| w_1 \| = 1, \tag{12}$$

$$u_1 = Xc_1 \quad \text{with} \quad \| c_1 \| = 1, \tag{13}$$

which create a linear relationship of parameters and steady states, respectively, meaning the first axis of the elements of $P_{q'\times s}$ and $X_{q'\times m}$. Meanwhile, for parameter sets, in order to predict steady state sets better, their principal components are required to have the largest covariance, i.e., $max\,\{cov(t_1, u_1)\}$.

After a tedious manipulation, we obtain:

$$P'XX'Pw_1 = \theta_1^2 w_1, \tag{14}$$

$$X'PP'Xc_1 = \theta_2^2 c_1, \tag{15}$$

where θ_i^2 $(i = 1, 2)$ is the maximum eigenvalue of matrices $P'XX'P$ and $X'PP'X$, w_1 and c_1 are their corresponding unit eigenvectors, respectively. At this point, their correlativity has been obtained and the foundations have been established as follows

$$P = t_1 q_1^T + E, \tag{16}$$

$$X = u_1 r_1^T + G, \tag{17}$$

$$X = t_1 b_1^T + F, \tag{18}$$

where E, F, and G are the corresponding residual matrices in regression equation. Besides, we gain the regression coefficient vectors q_1, r_1, b_1 and get the relationship between w_1 and q_1

$$q_1 = \frac{P^T t_1}{\| t_1 \|^2}, \tag{19}$$

$$r_1 = \frac{X^T u_1}{\| u_1 \|^2}, \tag{20}$$

$$b_1 = \frac{X^T t_1}{\| t_1 \|^2}, \tag{21}$$

$$w_1^T q_1 = w_1^T \frac{P^T t_1}{\| t_1 \|^2} = \frac{t_1^T t_1}{\| t_1 \|^2} = 1. \tag{22}$$

When the precision does not meet expectation, the residual matrices are used as new explanatory matrix and new response matrix until the cutoff condition is met and if $P_{q'\times s}$ extracts h components we obtain

$$P = t_1 q_1^T + t_2 q_2^T + \cdots + t_h q_h^T + E, \tag{23}$$

$$X = t_1 b_1^T + t_2 b_2^T + \cdots + t_h b_h^T + F. \tag{24}$$

Combining $w_i^T t_j = 1$ $(i = j)$ and $w_i^T t_j = 0$ $(i \neq j)$, and the matrix form can be expressed as

$$P = TQ^T + E, \tag{25}$$

$$X = TB^T + F = PWB^T + F = PA + F, \tag{26}$$

where $A = WB^T$.

The variable importance in projection (VIP) for parameter sets is calculated by:

$$p_{ij} = \sum_{k=1}^{h} t_{ik} q_{kj} + e_{ij}, \tag{27}$$

$$x_i = \sum_{k=1}^{h} t_{ij} b_k + f_i, \tag{28}$$

$$x_i = \sum_{k=1}^{h} p_{ij} w_{ik} b_k = \sum_{k=1}^{h} p_{ij} a_{jk}, \tag{29}$$

$$VIP_j = \sqrt{\frac{s \sum_{k=1}^{h} \left(b_k^2 t_k^T t_k \left(\frac{w_{jk}}{\|w_k\|} \right)^2 \right)}{\sum_{k=1}^{h} b_k^2 t_k^T t_k}}, \tag{30}$$

$$i = 1, \ldots, q', \quad j = 1, \ldots, s, \quad k = 1, \ldots, h,$$

where p_{ij}, x_i are the elements in parameter sets $P_{q' \times s}$ and PC1 of steady states $X_{q' \times m}$, respectively; t_k is the seleted PLS component; q' and s are the number and dimensionality of chosen parameter sets, h is the number of PLS components; q_{kj}, b_k, and a_{ij} defines the linear relationship between p_{ij}, t_k, and x_i, respectively. Besides, e_{ij}, and f_i are the residual errors of parameter sets and steady state sets. VIP scores are often used to identify the importance of each parameter in determining each cell fate.

7 Confidence ellipses

Table 4: Confidence ellipses of EMT.

Elliptical area	Ellipse center	Rotation angle	Semi-major axes	Semi-minor axes
Epithelial cell state	[-3.9903,0.1280]	2.1032	1.5015	0.5355
Mesenchymal cell state	[3.1315,-0.2931]	3.6518	3.3605	0.2023
Hybrid E/M cell state	[-0.1879,0.1354]	3.7209	2.3707	1.8682

Table 5: Confidence ellipses of ICM.

Elliptical area	Ellipse center	Rotation angle	Semi-major axes	Semi-minor axes
Epi -like state	[2.1334,-0.1201]	1.2775	3.1637	0.5573
PrE-like state	[-2.2691,0.2039]	1.3586	2.4737	1.1325
ICM-like state	[-0.2039,0.0034]	0.9465	1.8360	1.1000

8 PLSR coefficients

8.1 TS

Table 6: **PLSR coefficients. Constant terms:0.0600.**
Construct mapping of ten-dimensional parameters and PC1 of expression levels.

Parameters	g_a	k_a	$1/\lambda_a$	n_a	τ_a	g_b	k_b	$1/\lambda_b$	n_b	τ_b
PLS coefficient	-1.0114	0.7290	-0.2334	-0.2291	0.5071	0.9765	-0.7374	0.2482	0.2103	-0.5359

8.2 EMT

Table 7: **PLSR coefficients. Constant terms: -9.4958.**
Construct mapping when epithelial cells and mesenchymal cells are integrated.

Parameters	$T0$	$k0_T$	k_T	J_T	kd_T	$k0_s$	k_s	J_s	kd_s	k_S	J_S
PLS coefficient	12.9812	0.1225	0.8852	0.0682	-1.0939	0.0890	3.2998	-2.6164	-3.7871	2.9909	2.0555
Parameters	kd_S	$k0_3$	k_3	$J1_3$	$J2_3$	kd_3	$k0_z$	k_z	J_z	kd_z	k_Z
PLS coefficient	-3.0495	-1.1716	-1.1421	-0.6005	0.0936	1.7525	0.6077	1.2371	-1.9272	-1.5959	1.3903
Parameters	J_Z	kd_Z	$k0_2$	k_2	$J1_2$	$J2_2$	kd_2	k_{e1}	$J1_e$	k_{e2}	$J2_e$
PLS coefficient	0.9611	-1.3476	0.0333	-1.3954	-0.4149	-0.9494	2.0059	-0.1398	-0.1324	0.1182	0.1080
Parameters	kd_e	k_{n1}	$J1_n$	k_{n2}	$J2_n$	kd_n	n_t	n_s	n_z	n_{r2}	n_{r3}
PLS coefficient	0.3796	0.2515	-0.1280	0.2238	0.3414	-0.2321	-0.0458	-0.8699	0.5793	-0.6272	0.1231

Table 8: **PLSR coefficients. Constant terms: -4.0309.**
Construct mapping between the parameter sets and steady states belong to epithelial cells.

Parameters	$T0$	$k0_T$	k_T	J_T	kd_T	$k0_s$	k_s	J_s	kd_s	k_S	J_S
PLS coefficient	1.8587	0.1510	0.0313	0.0990	-0.1705	-0.0039	0.1456	-0.1817	-0.1123	-0.0179	0.0856
Parameters	kd_S	$k0_3$	k_3	$J1_3$	$J2_3$	kd_3	$k0_z$	k_z	J_z	kd_z	k_Z
PLS coefficient	0.0028	-0.0569	-0.7260	-0.0041	-0.0065	0.8177	0.1047	-0.0471	0.0859	-0.0625	-0.0157
Parameters	J_Z	kd_Z	$k0_2$	k_2	$J1_2$	$J2_2$	kd_2	k_{e1}	$J1_e$	k_{e2}	$J2_e$
PLS coefficient	0.0274	0.0018	-0.0329	-0.7491	-0.0065	0.0651	0.7681	-0.4000	-0.0094	-0.2409	-0.0208
Parameters	kd_e	k_{n1}	$J1_n$	k_{n2}	$J2_n$	kd_n	n_t	n_s	n_z	n_{r2}	n_{r3}
PLS coefficient	0.7160	-0.0377	-0.0789	-0.0161	0.0391	-0.0305	-0.1769	-0.1536	-0.0560	-0.1728	-0.2387

Table 9: **PLSR coefficients. Constant terms: -0.5638.**
Construct mapping between parameter sets and steady states belong to mesenchymal cells.

Parameters	$T0$	$k0_T$	k_T	J_T	kd_T	$k0_s$	k_s	J_s	kd_s	k_S	J_S
PLS coefficient	2.8604	0.1872	0.9846	0.0709	-1.1157	0.0192	2.6489	-1.2068	-2.8184	1.9922	0.5965
Parameters	kd_S	$k0_3$	k_3	$J1_3$	$J2_3$	kd_3	$k0_z$	k_z	J_z	kd_z	k_Z
PLS coefficient	-2.0567	-0.6685	-0.0223	-0.0326	-0.0279	0.7364	0.1678	1.1140	-1.3925	-1.2922	0.6098
Parameters	J_Z	kd_Z	$k0_2$	k_2	$J1_2$	$J2_2$	kd_2	k_{e1}	$J1_e$	k_{e2}	$J2_e$
PLS coefficient	-0.0172	-0.6510	-0.0259	0.1062	0.0508	-0.0423	-0.0051	-0.0075	-0.0268	0.0017	0.0043
Parameters	kd_e	k_{n1}	$J1_n$	k_{n2}	$J2_n$	kd_n	n_t	n_s	n_z	n_{r2}	n_{r3}
PLS coefficient	0.0733	0.4037	-0.0955	0.2517	0.0204	-0.7142	0.7460	-0.2790	0.3042	0.2778	0.6199

8.3 ICM

Table 10: **PLSR coefficients. Constant terms:7.3125 .**
Construct mapping when Epi-like and PrE-like types are integrated.

Parameters	vsg_1	vsg_2	vsn_1	vsn_2	$vsfr_1$	$vsfr_2$	vex	vsf	va
PLS coefficient	-0.9348	-0.7960	1.1526	1.1977	-1.3634	-1.2435	0.8681	0.1561	-2.1063
Parameters	vi	kdg	kdn	$kdfr$	kdf	Kag_1	Kag_2	Kan	$Kafr$
PLS coefficient	-0.9348	-0.7960	1.1526	1.1977	-1.3634	-1.2435	0.8681	0.1561	-2.1063
Parameters	Kaf	Kig	Kin_1	Kin_2	$Kifr$	Ka	Ki	Kd	Fp
PLS coefficient	-0.1214	-0.6849	0.8243	0.6384	-0.6533	1.0274	-1.5479	2.1564	-10.3871

Table 11: **PLSR coefficients. Constant terms:2.7868 .**
Construct mapping between the parameter sets and steady states belong to Epi-like phenotype.

Parameters	vsg_1	vsg_2	vsn_1	vsn_2	$vsfr_1$	$vsfr_2$	vex	vsf	va
PLS coefficient	0.0082	0.0092	0.7539	0.8800	-0.1578	0.0043	0.8496	0.1343	-0.0064
Parameters	vi	kdg	kdn	$kdfr$	kdf	Kag_1	Kag_2	Kan	$Kafr$
PLS coefficient	0.0237	0.0046	-1.6630	0.1592	-1.0206	0.0392	-0.0063	-0.0279	0.0110
Parameters	Kaf	Kig	Kin_1	Kin_2	$Kifr$	Ka	Ki	Kd	Fp
PLS coefficient	-0.5220	0.0349	-0.0214	0.0013	-0.1128	0.0218	-0.0494	0.0313	-0.2018

Table 12: **PLSR coefficients. Constant terms:-1.2565.**
Construct mapping between the parameter sets and steady states belong to PrE-like phenotype.

Parameters	vsg_1	vsg_2	vsn_1	vsn_2	$vsfr_1$	$vsfr_2$	vex	vsf	va
PLS coefficient	-0.5940	-0.5483	0.0389	-0.0110	-0.8127	-0.6729	0.8900	-0.0096	-0.4728
Parameters	vi	kdg	kdn	$kdfr$	kdf	Kag_1	Kag_2	Kan	$Kafr$
PLS coefficient	0.4802	1.1890	-0.0164	1.5443	-0.8875	0.0456	0.0080	0.0121	0.1391
Parameters	Kaf	Kig	Kin_1	Kin_2	$Kifr$	Ka	Ki	Kd	Fp
PLS coefficient	-0.0074	0.0072	0.1493	0.0686	-0.0126	0.3234	-0.2215	0.4339	-1.3721

References

[1] Huang, B. *et al.* (2017) Interrogating the topological robustness of gene regulatory circuits by randomization. *PLoS Comput. Biol.*, **13**, e1005456.

[2] Tian, X. *et al.* (2013) Coupled reversible and irreversible bistable switches underlying TGFβ-induced epithelial to mesenchymal transition. *Biophys. J.* **105**, 1079-1089.

[3] Bessonnard, S.*et al.* (2014). Gata6, Nanog and Erk signaling control cell fate in the inner cell mass through a tristable regulatory network. *Development*, 141(19), 3637-3648.

[4] Yamanaka, Y.*et al.* (2010). FGF signal-dependent segregation of primitive endoderm and epiblast in the mouse blastocyst. *Development*, 137(5), 715-724.

[5] Burdon, T.*et al.* (1999). Suppression of SHP-2 and ERK signalling promotes self-renewal of mouse embryonic stem cells. *Dev. Biol.*, 210(1), 30-43.

[6] Cheng, J. *et al.* (2017) Partial least squares regression (PLSR) applied to NIR and HSI spectral data modeling to predict chemical properties of fish muscle. *Food Eng Rev*, **9**, 36-49.

[7] Geladi, P. and Kowalski, B. (2017) Partial least-squares regression: A tutorial. *Analytica Chimica Acta*, **185**, 1-17.

[8] Boulesteix, A. and Strimmer, K. (2006) Partial least squares: a versatile tool for the analysis of high-dimensional genomic data. *Briefings in Bioinformatics*, **8**, 32-44.
